# Islands of Tuberculosis Elimination: An Evaluation of Community-Based Active Case Finding in North Sumatra, Indonesia

**DOI:** 10.3390/tropicalmed5040163

**Published:** 2020-10-26

**Authors:** Elvi S. Siahaan, Mirjam I. Bakker, Ratna Pasaribu, Amera Khan, Tripti Pande, Alwi Mujahit Hasibuan, Jacob Creswell

**Affiliations:** 1 Yayasan Menara Agung Pengharapan Internasional, Medan Johor 20211, Indonesia; elvi.siahaan@gmail.com (E.S.S.); ratnapasaribu@gmail.com (R.P.); 2 KIT Royal Tropical Institute, 1092 Amsterdam, The Netherlands; M.Bakker@kit.nl; 3 Stop TB Partnership, 1218 Geneva, Switzerland; amerak@stoptb.org; 4 McGill International Tuberculosis Center, Montreal, QC H4A 3J1, Canada; tripti.pande@mail.mcgill.ca; 5 Kepala Dinas Kesehatan Provinsi Sumatera Utara, Medan 20028, Indonesia; alwi.mujahit@gmail.com

**Keywords:** tuberculosis, active case finding, community outreach, Indonesia, key population

## Abstract

Community-based active case finding (ACF) is needed to reach key/vulnerable populations with limited access to tuberculosis (TB) care. Published reports of ACF interventions in Indonesia are scarce. We conducted an evaluation of a multicomponent community-based ACF intervention as it scaled from one district to nine in Nias and mainland North Sumatra. Community and health system support measures including laboratory strengthening, political advocacy, sputum transport, and community awareness were instituted. ACF was conducted in three phases: pilot (18 months, 1 district), intervention (12 months, 4 districts) and scale-up (9 months, 9 districts). The pilot phase identified 215 individuals with bacteriologically positive (B+) TB, representing 42% of B+ TB notifications. The intervention phase yielded 509, representing 54% of B+ notifications and the scale-up phase identified 1345 individuals with B+ TB (56% of notifications). We observed large increases in B+ notifications on Nias, but no overall change on the mainland despite district variation. Overall, community health workers screened 377,304 individuals of whom 1547 tested positive, and 95% were initiated on treatment. Our evaluation shows that multicomponent community-based ACF can reduce the number of people missed by TB programs. Community-based organizations are best placed for accessing and engaging hard to reach populations and providing integrated support which can have a large positive effect on TB notifications.

## 1. Introduction

Tuberculosis (TB) has existed for millennia yet is the leading infectious disease killer worldwide [1]. In 2018, a United Nations (UN) High-Level Meeting reaffirmed the global commitment to meeting previously established ambitious targets and strategies to end TB by 2030 and set out interim targets for 2022 on the numbers of people treated for TB [2]. Every year, it is estimated that 3 million people with TB are undetected and/or remain unnotified to National TB Programs (NTPs) globally [1]. Lack of accessibility and availability of TB services are major drivers for this [3]. Those who are missed are often members of key/vulnerable populations (i.e., miners, prisoners, elderly, people living with human immunodeficiency virus (HIV), or people in hard to reach areas) [4,5]. Various global initiatives have been launched in an attempt to reach all people with TB including the Global Fund’s Strategic Initiative on TB and the Find.Treat.All initiative which focus attention on reaching people with TB who are missed by routine services [6,7]. To reach more people with TB, active case finding (ACF) strategies focused outside traditional health facilities are needed [8,9,10].

Previous literature indicates that numerous approaches have been explored to identify and diagnose people with TB through ACF. A study in Ethiopia documented the high impact on TB case notifications by using existing health extension workers to perform numerous tasks, such as community-based screening, sputum collection and laboratory techniques (i.e., community-based slide fixing) [11,12]. In Cambodia and Myanmar there have been multiple efforts to reach people with TB using mobile chest X-ray units. [13,14,15,16]. In India, ACF encompassing the use of mobile vans as well as house-to-house screening by community workers on a massive scale has been conducted through Global Fund projects and more recently by the government’s ambitious initiatives [17,18]. Initiatives conducted outside of health facilities to reach individuals who are either not seeking care or are seeking care in the informal sector require a critical component of community mobilization and acceptance [19]. The inclusion of communities in such interventions to ensure access and local acceptance is crucial and, therefore, many ACF interventions are supported by community-based organizations. The Global Plan to End TB highlights the importance of increased involvement by the communities and civil society in the fight to end TB [4] and it is a critical part of the development of national strategic plans for TB [20]. 

Indonesia contributes to 8% of the global TB incidence, making it the third highest burden country after India and China [1]. In 2018, an estimated 845,000 people developed TB and only 563,879 (67%) were notified, meaning that 275,000 (33%) people with TB were missed [1]. Indonesia’s health system is decentralized, and district health offices have the responsibility of organizing public health services through local facilities at the sub-district level called *Puskesmas* [21]. Encouraging ACF and community engagement in the TB response is one of the main strategies in Indonesia’s National Strategic Plan [22]. 

TB patient-pathway analyses from Indonesia documented that more than 67% of symptomatic TB patients initially sought care in the private sector. The analyses highlighted the importance of ‘community referrals’ within the pathway [21]. However, there is limited published literature on ACF interventions in Indonesia. One study compared contact investigation and door-to-door screening by community health workers (CHWs) in Bandung City. The authors concluded that CHWs can be used to improve acceptance by the community, however no people with TB were detected in the study [23]. A modeling study comparing three case finding strategies concluded that if ACF is used to lower the proportion of people not accessing care, it can reduce mortality [24]. Due to the paucity of literature on ACF in Indonesia and the need to improve case detection, we report on the results of an evaluation of an ACF intervention in Nias archipelago and mainland North Sumatra, Indonesia funded by Stop TB partnership’s TB REACH initiative [25]. 

## 2. Materials and Methods 

### 2.1. Setting

We conducted an evaluation of scaled ACF interventions implemented in North Sumatra province. North Sumatra is the fourth most populous province in Indonesia consisting of 419 islands. The ACF intervention was conducted on Nias archipelago and mainland North Sumatra. Nias is an archipelago off the western coast of Northern Sumatra consisting of 32 inhabited islands. Many of the islands are difficult to reach and are only accessible by boat. The total population of the archipelago is approximately 800,000 people [26]. Nias is one of the poorest areas in Sumatra, and many of the indigenous residents are illiterate and do not speak Bahasa, limiting their access to official health information. Additionally, access to healthcare in Nias is often limited due to distances, high staff turnover, and lack of funding and training for healthcare staff in these remote areas. For much of the project period, Nias had only one GeneXpert machine which was mostly non-functional. Mainland North Sumatra is more developed than Nias. The total population is approximately 3 million people. Despite better access to health services compared to Nias, North Sumatra has a medium ranking for its health development index indicators (education, life expectancy and per capita income) in most districts [27]. The districts on the mainland each had a single GeneXpert machine which was used primarily to test for drug resistance rather than diagnosis. Yayasan Menara Agung Pengharapan Internasional (YMAPI), a local non-governmental organization based in North Sumatra has provided access to health services and medicine for more than 15 years. YMAPI was the main implementer of this intervention, working in collaboration with the local District Health Offices and the NTP.

### 2.2. Timeline and Coverage

**Figure 1:**
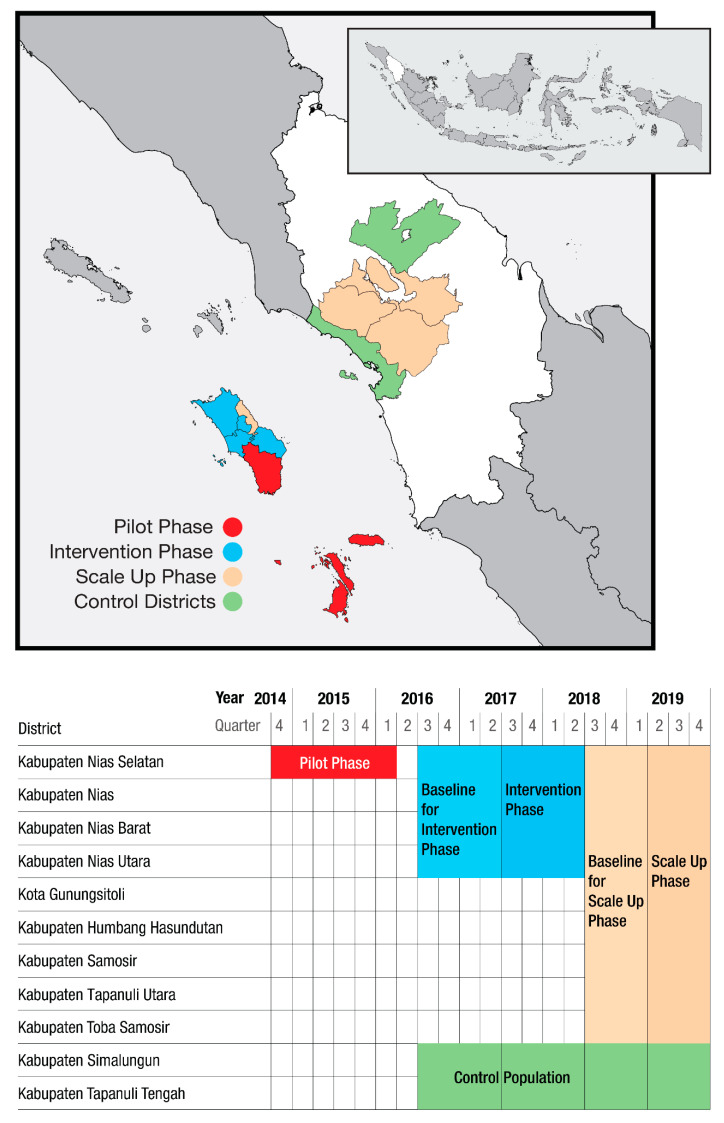
Active case finding in North Sumatra—timelines and geographic areas.

This community-based ACF intervention was conducted in three phases. Figure 1 presents a map of the area and timeline for the intervention and control districts. A pilot phase began in October 2014 in Nias Selatan District (population approximately 182,000) to test the intervention and help train project staff, lasting through March 2016. The ACF intervention phase (hereafter intervention phase) took place in four of the five districts within Nias (population approximately 450,000) between July 2017 and June 2018. Between April and December 2019, the ACF scale-up phase (hereafter scale-up phase) was implemented in all five districts on Nias as well as four additional districts in the mainland North Sumatra (population approximately 740,000). Two purposefully selected districts in North Sumatra with stable notifications rates and lacking other case detection interventions were used as control districts as part of TB REACH’s standard monitoring and evaluation methodology [28]. Throughout the different areas and across the phases, the ACF interventions were similar but lessons learned from earlier phases were incorporated during implementation of subsequent phases. 

### 2.3. Community-Based Outreach Intervention

**Figure 2:**
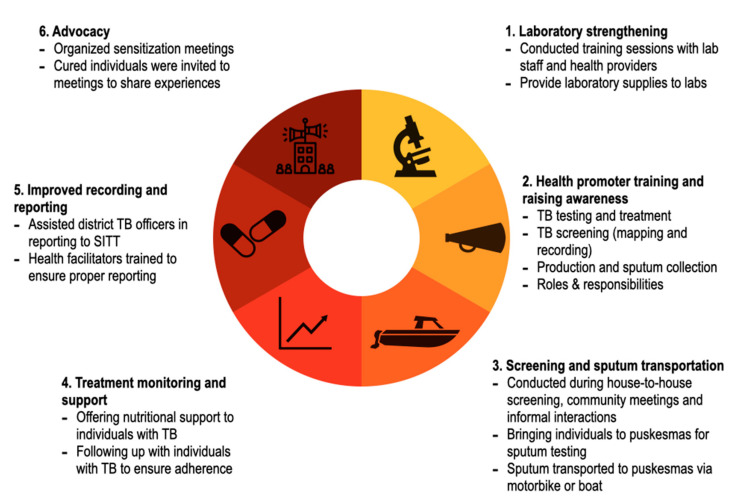
Multicomponent community-based active case-finding intervention in North Sumatra, Indonesia.

A multicomponent community based ACF intervention was developed in coordination with the NTP and was conducted as part of the TB programme operations (see Figure 2). Predominantly female community-based volunteers (health promoters) who lived and worked in the communities were the core of this intervention. There were 1505 health promotors engaged in the pilot phase; 3730 in the intervention phase; and 7835 during the scale-up phase. In the pilot and intervention phase, health promotors were selected by the head of the different villages and other community leaders, while during the scale-up phase existing CHW (*posyandu kader*) were selected. The health promoters did not receive a salary but were provided small in-kind support such as transport reimbursements, T-shirts, caps, notebooks, and an official inauguration ceremony with a certificate. Health promoters were trained and supervised by project staff (health facilitators) to raise TB awareness, to sensitize community members on the importance of TB diagnosis and treatment and to screen community members for signs and symptoms of TB. The health promotors also advocated for health-seeking behaviors and provided information on nutrition, sanitation, and the harmful effects of tobacco use. The health facilitators, who received a small salary, travelled to hard-to-reach areas and villages by foot, bicycle, motorbike, and/or boat to support the screening and referral of people with presumptive TB to link them to testing, diagnosis and treatment.

The intervention targeted people at the village level. The health promoters and facilitators disseminated their health awareness messages and conducted TB screening during house to house visits and informal meetings where people congregated such as town meetings and events. A primary focus of their work was to promote the value of the local *Puskesmas* in providing high-quality medicine and care for TB as confidence in the health system was perceived as low. 

Individuals were screened verbally for seven symptoms. The symptoms included: cough for more than 2 weeks, weight loss, loss of appetite, difficulty breathing, prolonged fever, night sweats and coughing blood. Anyone reporting two or more symptoms was considered to have presumptive TB and was eligible for diagnostic testing. All individuals with presumptive TB were asked to provide two sputum samples. In most situations, a health promoter or health facilitator accompanied them to the nearest *Puskesmas* for testing. A small enabler was provided to support the travel. For individuals living far from the *Puskesmas*, a health facilitator collected samples in the village and transported them to the nearest laboratory using a cold box by motorcycle and/or boat. In some instances, in very remote islands, laboratory technicians visited the communities, collected sputum and fixed slides on site. All diagnostic testing was done with sputum microscopy and individuals were eligible to initiate treatment if one of the smear results was positive/scanty in accordance with NTP guidelines. All individuals with TB were provided treatment support through the health facilitators and were also offered nutritional support consisting of food packages.

To ensure the ACF activities did not overwhelm the health system’s ability to provide care, YMAPI procured laboratory equipment including 17 microscopes and supplies for 85 diagnostic facilities in the intervention areas. Training sessions for laboratory and facility staff on screening, diagnostics and laboratory procedures, and treatment were also provided. People initiating TB treatment were notified through the TB registers as per standard NTP practice. YMAPI also worked with district TB officers to ensure timely and accurate reporting in the SITT (*Sistem Informasi Terpadu TB*), the national TB reporting system. 

Finally, the project organized sensitization and results sharing meetings for village leaders, district heads and government staff. In these meetings people with TB currently on treatment or those who had completed their treatment were invited to participate to share their stories and advocate for more local funding for the TB program (see Figure 2).

### 2.4. Data Collection 

Quarterly notification data from the SITT from the district and provincial offices from October 2013 to December 2019 were extracted. If online reporting data were incomplete, we complemented the reports with facility level data directly from the *Puskesmas*. The numbers of people screened and identified as having presumptive TB were collected by the health facilitators directly from the health promoters and then tracked into the facility laboratory registers to determine yield. 

### 2.5. Data Analysis

Evaluation of the ACF intervention followed the standard monitoring and evaluation framework of TB REACH to determine the impact of case finding in a given area [28]. For all three phases, official NTP notification data were analyzed using a pre-post evaluation methodology of bacteriologically positive (B+) TB notifications in intervention areas. In addition, a control area was used for the intervention and scale-up phases. We compared the notifications in a baseline period to the notifications during the ACF intervention and did the same in control districts. Since the pilot phase lasted 6 quarters, for the baseline data we multiplied the four previous quarters of notifications by 1.5 to get a comparable result. Other periods all used actual notification numbers. We calculated the percentage change in TB notifications between baseline and intervention periods, as well as the absolute number of additional people notified. In addition, project data were used to track indicators relating to the number of presumptive cases among screened and the yield of testing during the ACF intervention to complement the results of the change in TB notifications.

### 2.6. Ethics Statement

This intervention was approved by the District Administration as part of programmatic services thus no additional ethical approval was required. All patient information was anonymized, and only aggregate data were used in the analyses.

## 3. Results

During the 18-month pilot phase (Q4 2014–Q1 2016), ACF in one district (Nias Selatan) was conducted. This involved five of the 11 Puskesmas with TB testing facilities and three Satellite Puskesmas. As shown in Table 1, during the pilot phase, health facilitators identified 3261 people who had presumptive TB, and were able to ensure 2983 (91.5%) were tested by linking them to laboratory services. Data on numbers of people screened were not collected in a systematic manner during the pilot phase. The ACF yielded 215 people with B+ TB (7.2% positivity rate), all of whom initiated treatment. Overall, there were 509 people with B+ TB notified in Nias Selatan during this phase, meaning community outreach efforts were responsible for 42% of the total B+ notifications during the pilot period. The computed 18-month B+ TB notifications prior to the pilot phase in Nias Selatan were 444, signifying a 15% increase using a pre/post analysis (Table 2). 

During the 12-month intervention phase (Q3 2017–Q2 2018) outreach was conducted in four districts in Nias. Health facilitators verbally screened 124,430 individuals. There were 6084 individuals who screened positive and were referred for testing (4.9% of those screened). The vast majority of individuals with presumptive TB, were tested (5807 or 95.4%). Of those tested, 509 (8.8%) had B+ results and 492 (96.7%) of them initiated anti-TB treatment (Table 1). Total B+ notifications during the intervention phase in the evaluation area, including passive case finding, was 916, indicating that community based ACF contributed 54% of B+ notifications. In the four quarters prior to the intervention phase, there were 495 B+ notifications in the same districts meaning that B+ TB notifications increased 85% in the four intervention districts. At the same time, we observed a modest increase in B+ TB notifications in the control population during the intervention phase, moving from 1424 to 1653, (+16%) (Table 2). 

The scale-up phase included an additional district on Nias and four districts on mainland North Sumatra meaning a total of nine districts were covered. During the scale-up phase the number of people screened was 252,774 and the numbers were similar between Nias and the mainland. Of those screened, 9744 (3.9%) were referred for testing and 8962 symptomatic individuals (92.0%) were tested. Higher rates of presumptive TB were found on Nias compared to the mainland (4.3% vs. 3.4%), and the proportion tested among those referred was also higher on Nias (96.8% vs. 85.9%). Among people identified by the ACF who were tested, 823 (9.2%) had B+ results and 758 (92.1%) initiated treatment. Pretreatment loss to follow-up was slightly higher on Nias (9.6% vs. 8.3%). Across all the nine districts, there were 1345 B+ TB notifications during the scale-up phase meaning that the ACF activities identified 56% of the total B+ cases notified. ACF in districts on Nias contributed slightly more than the yield on the mainland (58% vs. 55%). B+ notifications on Nias island continued to rise compared to the baseline period (+22%), while on the mainland there was almost no change (−2%). When the change by district on the mainland was evaluated, we noted a wide range from an increase of 130% in Kabupaten Humbang Hasundutan, to a decrease of 41% in Kabupaten Tapanuli Utara, but the overall change was minimal. The control population had B+ notifications that also remained almost unchanged (+1%) with no variation between the control districts. Overall, the outreach activities screened 377,204 people, and tested 17,752 for TB, identifying 1547 people with B+ TB, and linking 94.7% of them to treatment. 

## 4. Discussion

To our knowledge, our results are the first published account of large-scale ACF for TB in Indonesia. Our results show that combining health system strengthening, community mobilization, and ACF activities reached 1547 people with B+ TB and linked 95% of them to treatment. Despite a longstanding focus on bringing basic services to people with TB, our evaluation suggests many people in Indonesia with TB need additional measures to reach them. Previously, ACF has been used in numerous settings with different outreach models [11,12,13,14,15,16,17,18,29,30,31,32]. While Indonesia has a strong private sector where many people with TB seek care [22], evidently there are places, especially in remote rural areas, where community-based approaches are needed to reach people with TB. 

ACF is often effective in situations where there is poor access to care due to stigma, travel times, distances, and/or cost barriers [15,31,32]. Although we did not measure the impact of this intervention on out-of-pocket costs for people with TB, ACF has been shown to reduce catastrophic costs for people with TB [33]. In addition, ACF reaches people earlier in their disease progression, although this has not translated into improved treatment outcomes, it may lessen individual suffering [34]. 

We believe our intervention was successful, not only because of the outreach efforts for screening of villagers, but because of the multifaceted approach that was taken including supporting public health facilities with laboratory supplies and political advocacy. While ACF found more than 50% of the total TB notifications during the intervention, the numbers of diagnostic tests undertaken were also very large. Identifying individuals with presumptive TB and getting their samples to laboratories through the provision of enablers and a transport system for sputum was critical [35]. To identify more people with TB, large numbers of people must be tested. In Nigeria and India, ACF studies demonstrated these increases in testing were necessary to generate gains in notifications [36,37]. ACF efforts can place an enormous burden on the laboratory system and this was one of the main reasons to also strengthen the infrastructure and the capacity of laboratories to diagnose TB. 

In addition to the community-based screening, the intervention focused on improving health information and the promotion of health-seeking behavior in the communities. It also supported the district TB officers to improve recording and reporting and provided political advocacy for local government to provide more support for the health services in the area. These comprehensive actions were aimed at strengthening the overall health services and promoting the accessibility to the services within the community for longer-term sustainability [38]. 

While the ACF intervention increased the numbers of people detected with TB, national disease estimates suggest people with TB are still being missed. In our evaluation, the rates of bacteriologically negative TB (clinically diagnosed) overall were low (~24%), and in the first two phases it was only 10% of all forms (AF) notifications. On North Sumatra there were higher proportions, up to 55% (data not shown). On Nias and its surrounding islands it remains difficult to make a clinical diagnosis of TB at the *Puskesmas* level as there is only one X-ray machine available on the island. Presumptive individuals must be referred and bear the cost of the X-ray which is a disincentive. Additionally, we only used symptom screening to identify presumptive TB. Multiple prevalence surveys across Asia have shown that confining testing to only symptomatic people will miss a large proportion (40–79%) of people with TB [39]. Since we did not have access to X-ray services, people with TB were probably missed. Finally, it is well known that the sensitivity of smear microscopy is poor [40,41]. Ideally Xpert MTB/RIF testing would be used in ACF situations. However, despite large investments in Xpert in Indonesia with more than 500 machines procured as of 2017 [42] and efforts to expand testing, access to diagnostic Xpert testing on Nias Island and Sumatra was very low. Ensuring testing for all people with presumptive TB is a challenge globally; while it is recommended in many national guidelines, only a few countries are able to provide access to rapid molecular tests for initial diagnosis [21]. Expanding access to testing should be strongly considered to conform to World Health Organization (WHO) recommendations [43] and to identify more people with bacteriologically confirmed TB [44,45].

While the ACF contributed to large increases in B+ notifications on Nias, the same activities had a variable impact on mainland North Sumatra. We are not sure why these results were different but have some hypotheses. The interventions were implemented on the mainland for a short period of time, which did not permit the same level of collaboration with local authorities, a factor that is hard to measure, but we feel is important to community-based work. Mainland North Sumatra is also more developed and access to diagnostic and treatment facilities is better than on Nias. We did see variation between the districts on the mainland with the most developed districts showing a decrease in notifications while the less-developed districts had an increase. ACF will not perform the same in all areas and for all populations and thus tailoring the outreach to address local barriers is critical.

Limitations of this intervention include the fact that neither the intervention nor control areas were randomly selected, and by using as baseline for the scale-up phase in Nias the three quarters directly following the intervention phase, we may have slightly underestimated the effect, as some of the sensitization activities can be expected to have a lasting effect beyond the intervention itself. In addition, we were not able to use molecular diagnostic tests, culture, nor X-ray due to the resources available in the project areas. These tools would have likely helped identify more people with TB, however the reality in many countries is that access to modern tools of TB diagnosis are often limited to well-equipped urban areas [46]. Since smear microscopy and Xpert are not 100% specific there is a possibility that a proportion of the sputum smear positive individuals may have false positive results, a risk that all ACF interventions where positivity rates are low must consider [47]. The laboratory positivity rate in our interventions was close to 9%, which is actually higher than documented yields in some TB programs and similar to passive case finding [48,49]. As we describe the results of an evaluation of a specific programmatic intervention, the impact of a similar approach in other parts of Indonesia or other countries may not be generalizable. However, with the growing body of evidence around the impact of ACF interventions [50] we believe there are likely many areas where remote rural populations could benefit from similar activities. Our results are from a programmatic implementation, not a controlled research experiment, limiting the data we can collect and conclusions we can draw, but also providing a better understanding of what is feasible in a ‘real world’ situation. 

## 5. Conclusions

Despite a well-established TB program, there are many poor and remote communities where access to health services is lacking in Indonesia. By combining community-based education and outreach with training and infrastructure support to health services and political advocacy, large numbers of people with TB can be reached. These comprehensive types of intervention should be considered in other areas with deficient access to care. Expanding the screening approach to include both X-ray and Xpert to identify asymptomatic cases through better screening and enhance diagnostic sensitivity would likely improve results even more.

## Figures and Tables

**Table 1:** Active case finding intervention data from all phases, North Sumatra.

	Pilot Phase ^#^	Intervention Phase ^+^	Scale-up Phase ^&^			Overall Total
			Nias	Mainland	Total	
People Screened	N/A	124,430	126,384	126,390	252,774	377,204
People Referred for Testing	3261	6084	5464	4280	9744	19,089
People Tested	2983	5807	5287	3675	8962	17,752
People with B+ TB	215	509	429	394	823	1547
People Initiated on Treatment	215	492	388	370	758	1465
Presumptive Rate (Presumptive/Screened)		4.9%	4.3%	3.4%	3.9%	4.2% **
% of presumptive people tested	91.5%	95.4%	96.8%	85.9%	92.0%	93.0%
% yield of TB testing	7.2%	8.8%	8.1%	13.1%	9.2%	8.7%
% B+ linked to treatment	100.0%	96.7%	90.4%	91.7%	92.1%	94.7%

TB: tuberculosis; N/A: data unavailable, B+: bacteriologically positive. ^#^ Pilot Phase included 1 district (Q4 2014–Q1 2016). ^+^ Intervention Phase included 4 districts on Nias Island (Q3 2017–Q2 2018). ^&^ Scale up Phase included 9 districts total (5 districts on Nias Island and 4 districts on mainland North Sumatra; Q2 2019–Q4 2019). Diagnosis was through sputum smear microscopy. ** Includes only data from active case finding (ACF) Intervention phase and Scale-up phase since pilot phase did not track screening numbers.

**Table 2:** Active case finding yield and impact on bacteriologically positive TB notifications, North Sumatra.

	B+ TB Notifications Intervention Districts	B+ TB Notifications Control Districts
Pilot phase Nias *		
Baseline ^	444	NA
Intervention	509	NA
ACF direct yield	215	NA
% notifications identified by ACF	42%	NA
Number and (%) change from baseline	65 (+15%)	NA
**Intervention phase Nias ^+^**		
Baseline	495	1424
Intervention	916	1653
ACF direct yield	492	NA
% notifications identified by ACF	54%	NA
Number and (%) change from baseline	421 (+85%)	229 (+16%)
**Scale-up phase Nias ^&^**		
Baseline	551	1176
Intervention	673	1184
ACF direct yield	388	NA
% notifications identified by ACF	58%	NA
Number and (%) change from baseline	122 (+22%)	8 (+1%)
**Scale-up phase Mainland North Sumatra ^&^**		
Baseline	686	1176
Intervention	672	1184
ACF direct yield	370	NA
% notifications identified by ACF	55%	NA
Number and (%) change from baseline	−14 (−2%)	8 (+1%)

Notifications only include people who initiated anti-TB treatment. * Pilot Phase included 1 district (Q4 2014–Q1 2016). ^ Baseline in pilot phase includes 4 quarters of notification numbers prior to pilot phase (*n* = 296) multiplied by 1.5 (for 6 quarters, *n* = 444) since the intervention in the pilot phase lasted 6 quarters. ^+^ ACF Intervention Phase included 4 districts on Nias Island (Q3 2017–Q2 2018). ^&^ Scale up Phase included 9 districts total (5 districts on Nias Island and 4 districts on mainland. North Sumatra; Q2 2019–Q4 2019). ACF = active case finding. B+ = bacteriologically positive.
